# Effects of Co-Exposure of Nanoparticles and Metals on Different Organisms: A Review

**DOI:** 10.3390/toxics9110284

**Published:** 2021-11-01

**Authors:** Yasmina M. Abd-Elhakim, Mohamed M. Hashem, Khaled Abo-EL-Sooud, Bayan A. Hassan, Khlood M. Elbohi, Adham A. Al-Sagheer

**Affiliations:** 1 Department of Forensic Medicine and Toxicology, Faculty of Veterinary Medicine, Zagazig University, Zagazig 44511, Egypt; kmelbohy@zu.edu.eg; 2 Department of Pharmacology, Faculty of Veterinary Medicine, Cairo University, Giza 12211, Egypt; hashemm41@cu.edu.eg (M.M.H.); kasooud@cu.edu.eg (K.A.-E.-S.); 3 Pharmacology Department, Faculty of Pharmacy, Future University, Cairo 41639, Egypt; Bayan.saffaf@fue.edu.eg; 4 Department of Animal Production, Faculty of Agriculture, Zagazig University, Zagazig 44511, Egypt

**Keywords:** engineered nanomaterials, heavy metals, bioaccumulation, environment, co-exposure, mixture toxicity

## Abstract

Wide nanotechnology applications and the commercialization of consumer products containing engineered nanomaterials (ENMs) have increased the release of nanoparticles (NPs) to the environment. Titanium dioxide, aluminum oxide, zinc oxide, and silica NPs are widely implicated NPs in industrial, medicinal, and food products. Different types of pollutants usually co-exist in the environment. Heavy metals (HMs) are widely distributed pollutants that could potentially co-occur with NPs in the environment. Similar to what occurs with NPs, HMs accumulation in the environment results from anthropogenic activities, in addition to some natural sources. These pollutants remain in the environment for long periods and have an impact on several organisms through different routes of exposure in soil, water, and air. The impact on complex systems results from the interactions between NPs and HMs and the organisms. This review describes the outcomes of simultaneous exposure to the most commonly found ENMs and HMs, particularly on soil and aquatic organisms.

## 1. Introduction

Engineered nanomaterial (ENMs) have distinctive physical and chemical features, e.g., rapid diffusion, large surface areas, liquid or gas-phase reactivity, and small sizes [1]. Numerous uses of ENMs have been found to date for commercial products, building materials, agriculture, electronics, and pharmaceuticals [2,3,4]. Global ENMs production, estimated at 58,000 tons a year as of 2020, will result in a significant environmental release of nanoparticles (NPs) [5,6]. Because the manufacturing of diesel cars, motorcycles, and metallurgical emits NPs into the air [7], their occurrence in the environment will affect the quality of life [8].

During the last decade, many studies on the toxicity of NPs have been conducted. The exposure of living organisms to NPs or NPs/pollutants adducts can induce adverse results in various physiological systems [9,10]. In most studied NPs, their toxicity was associated with oxidative stress caused by reactive oxygen species (ROS) [11,12,13,14].NPs may also display toxicity by reacting with biological macromolecules or releasing toxic components, such as metal ions [15,16,17,18,19].

It is unlikely that no other toxic species co-exists with ENMs in a realistic environment. Thus, the emitted NPs can interact with pre-existing pollutants, resulting in bioaccumulation and/or toxicity. Thus, the outcomes of interactions between NPs and co-existing contaminants have become a rising issue of scientific investigation [20]. For instance, the water and soil contamination by heavy metals (HMs), such as cadmium (Cd), arsenic (As), lead (Pb), nickel (Ni), and copper (Cu), has become a serious global environmental problem because of the increased anthropogenic and industrial activities [21,22,23,24]. There are several co-exposure scenarios of commonly used NPs and widely spread HMs. For example, titanium dioxide NPs (TiO_2_NPs) and Cd can co-exist in different systems. This is due to TiO_2_NPs release from wastewater treatment plant effluents to freshwater bodies [25,26], and Cd^+2^ has been described as a concern [27]. In parallel, the use of TiO_2_NPs as nano-fertilizer and nano-pesticide [28,29] could also raise concerns due to the fact that Cd^+2^ is a soil primary contaminant [30,31,32,33].

The potential interaction between NPs and HMs inside the living organism could differ with the type of NPs. For instance, Yu*,* et al. [34] assessed the effects of four commonly used NPs, including TiO_2_, silica oxide (SiO_2_), silver (Ag), and CdTe/CdS core/shell quantum dots (QD) on the Cd^+2^ toxicity to the freshwater algae *Chlamydomonas reinhardtii*. Their results demonstrated that both SiO_2_NPs (400 mg L^−1^) and TiO_2_NPs (100 mg L^−1^) diminished the Cd^+2^ toxicity to *C. reinhardtii*. On the other hand, QD (0.5 mg L^−1^) augmented the Cd^+2^ toxicity to algae, while no significant change was detected in the toxicity of Cd^+2^ to algae with combined exposure to Ag NPs (0.2 mg L^−1^). Therefore, this review discusses the reported co-exposure outcomes between ENMs and HMs in the different in vivo and in vitro experimental models (Table 1).

## 2. Titanium Dioxide Nanoparticles

The TiO_2_NPs are currently among the most commonly used NPs in a significant number of consumer products, such as cosmetic products, sunscreens, dyes, catalysts, food colorings, and biomedical applications [63,64,65]. The global output of TiO_2_NPs estimated was about 5000 tons per year in 2006–2010 and 10,000 tons annually in 2011–2014, with an expected output of 2.5 million tons per year by 2025 [66]. TiO_2_NPs have a wider surface-to-volume ratio than conventional TiO_2_ and provide increased adsorption potentials for co-existing pollutants via sorption [67].

Engates and Shipley [68] have shown that TiO_2_NPs had high adsorption rates for several HMs, including Cd^+2^, Pb^+2^, Zn^+2^, Cu^+2^, and Ni^+2^. Additionally, Zhang*,* et al. [37] reported that As is more sorbed to the TiO_2_NPs than the sediment particles. Furthermore, Li*,* et al. [69] and Deng*,* et al. [70] demonstrated that TiO_2_NPs facilitated the contaminants uptake into aquatic organisms, including fish and crustaceans, through the adsorption to NPs surfaces. The bioaccumulation and toxicity of Pb^+2^, Cd^+2^, Zn^+2^, As^+3^, and Cu^+2^ to zebrafish (*Danio rerio*) larvae, carp (*Cyprinus carpio*), and *Daphnia magna* augment significantly due to TiO_2_ NPs [40,43,47,52,55]. However, it has been reported that the Cd^+2^ and Cu^+2^ toxicity, in the presence of TiO_2_NPs, decreased in algae (*Chlamydomonas reinhardtii* and *Microcystis aeruginosa*) and amphipod (*Gammarus fossarum*) due to their reduced bioavailability [53,71,72]. In addition, As^+5^ toxicity to the freshwater flea *(Ceriodaphnia dubia)* may be worsened or eliminated by TiO_2_NPs, depending on the ratio of TiO_2_NPs to As^+5^ [48]. Species-defined interactions with TiO_2_NPs and HMs, differences in model organisms, and physicochemical parameters of the co-exposition medium may be underlying these conflicting effects.

**Figure 1:**
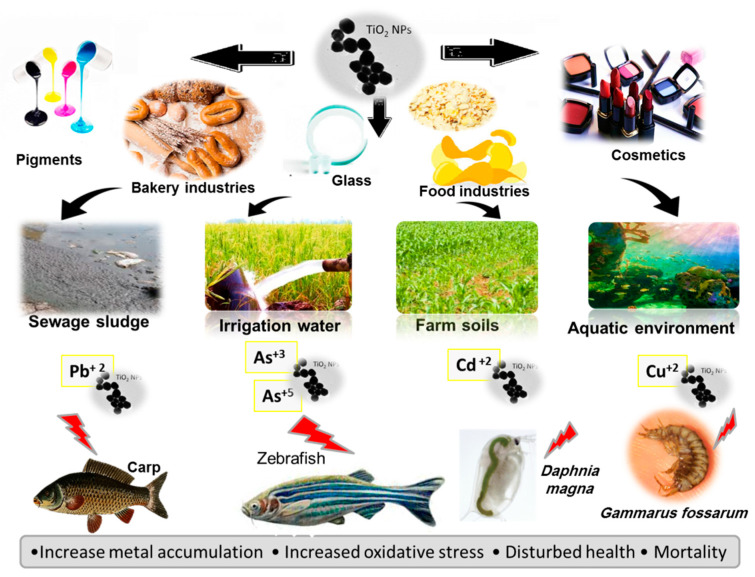
Sources of titanium dioxide nanoparticles exposure, possible interactions with heavy metals, and negative effects on different organisms.

Wang*,* et al. [73] evaluated the TiO_2_NP (5 and 15 nm) effect on the Cd^+2^, As^+3^, and Ni^+2^ bioaccumulation and toxicity in *Caenorhabditis elegans* during the sedimentation process in the aquatic environment. The authors verified that the HMs hastened the aggregation of TiO_2_NPs. The TiO_2_NPs quick aggregation and sedimentation altered the vertical distribution of HMs through adsorption and prolonged benthic species exposure. The main factors affecting the sedimentation rate were aggregate particle size and ion strength. TiO_2_NPs at non-toxic levels competently increased the HMs bioaccumulation and reproductive toxicity to *C. elegans* in a dose- and size-dependent manner; however, the TiO_2_NP effect on As^+3^ was lower than that on Cd^+2^ and Ni^+2^. These data demonstrate clearly that TiO_2_NPs potentiate the HMs toxicity to *C. elegans* due to their increased accumulation in the presence of TiO_2_NPs. Importantly, the interactions and fate of TiO_2_NPs and HMs that occur during sedimentation and the responses in organisms induced by co-exposure should be considered a necessary and integral part of the ecological system risk assessment (Figure 1).

### 2.1. TiO_2_NPs and Cd Co-Exposure

The TiO_2_NP release into water bodies is highly predictable with the increased use for these ENMs [74]. At the same time, Cd^+2^ is the most predominant water pollutant [75]. The TiO_2_NPs and Cd^+2^ may be released into the same freshwater system, affecting the contaminants’ mode of action and fate. Recent studies confirmed the high ability of TiO_2_NPs to adsorb Cd, hasten its transport rate into host tissues, and increase its concentration within the tissues [37,41,42].

Zhang*,* et al. [37] reported that, in the presence of TiO_2_NPs, Cd^+2^ concentrations increased by 146% in carp, and a positive link was identified between Cd^+2^ and TiO_2_NPs levels. In addition, in the viscera and gills of carp, considerable Cd^+2^ and TiO_2_ accumulated.

Balbi*,* et al. [38] evaluated the effects of TiO_2_NPs and Cd^+2^ co-exposure on *Mytilus galloprovincialis*, a marine bivalve. Cd^+2^ suppressed the TiO_2_NPs-induced increase in immune parameters, including lysozyme activity and nitric oxide production in the hemolymph. The TiO_2_NPs and Cd^+2^ interaction in the digestive glands affected various lysozyme indicators, including the accumulation of lipid, stability of the lysosomal membrane, and lysosome/cytoplasm volume ratio. In addition, the expression of immune-related genes encoding lysozyme-and toll-like receptors was altered due to TiO_2_NPs and Cd^+2^ co-exposure. However, TiO_2_NPs did not alter the induction of metallothionein or Cd^+2^ accumulation in the analyzed tissues. Hence, the authors verified that the modifications in Cd^+2^ bioavailability or bioaccumulation in the presence of TiO_2_NPs were not responsible for interactive effects detected on different estimated indicators. Such effects could result from interacting with one contaminant at the different biological organizations, with both common and distinct targets/action mechanisms. Generally, their findings showed that the TiO_2_NPs and Cd^+2^ mutual exposures did not increase the negative impact on *M. galloprovincialis*.

The single or joint effects of TiO_2_NPs (21 nm) and humic acid (HA) on Cd^+2^ bioaccumulation in zebrafish were investigated by Hu*,* et al. [39]. They found that TiO_2_NP (5–20 mg/L) in HA-containing water could change the effects of exposure of zebrafish to Cd^+2^ and other probable HMs. There is no clear mechanism underlying these combined effects.

Hartmann*,* et al. [41] evaluated the Cd toxicity to two freshwater organisms, *Daphnia magna* and oligochaete *Lumbriculus variegatus*. Results showed that the total body burden and Cd^+2^ toxicity to *L. variegatus* were unaffected by TiO_2_NPs exposure, showing that Cd^+2^ adsorption to TiO_2_NPs did not disturb total bioavailability. In addition, in *D. magna*, no change in toxicity was observed despite facilitated Cd^+2^ uptake by TiO_2_NPs and increased total body burden of Cd^+2^.

Yang*,* et al. [42] investigated the TiO_2_ NPs effect on Cd^+2^ bioavailability and toxicity to *Chlamydomonas reinhardtii* green algae. They reported that Cd^+2^ toxicity to green algae cells was reduced in the presence of TiO_2_NPs. In addition, no measurable TiO_2_NP amount was found to be associated with the algal cells. Hence, the authors demonstrated that TiO_2_NPs could decrease the free Cd^+2^ concentration, reducing its bioavailability and toxicity to *C. reinhardtii*. Furthermore, the electrostatic and potentially steric repulse between TiO_2_NPs and algal cells may interfere with their direct contact and prevent TiO_2_NPs from being internalized into the cells.

In the study of Tan and Wang [43], the absorption efficacy, aqueous uptake, and Zn^+2^ and Cd^+2^ toxicity were investigated after exposure of freshwater zooplankton, *Daphnia magna*, to 1 mg/L TiO_2_NPs for 2 days. The authors verified that *D. magna* pre-exposure to TiO_2_NPs resulted in a marked increase in Zn^+2^ and Cd^+2^ uptake from the dissolved phase. In addition, the metallothioneins and ROS measurements proved that the TiO_2_NPs provide potential adsorption binding sites for Zn^+2^ and Cd^+2^ within the *D. magna* gut.

Vale*,* et al. [45] evaluated the role of TiO_2_NPs on Cd^+2^ (112 μg/L) biouptake and toxicity for *Corbicula fluminea*, a freshwater bivalve. The authors confirmed that Cd-uptake by *C. fluminea* were not affected by TiO_2_NP presence.

Yang*,* et al. [44] found that TiO_2_NPs increased Cd^+2^ accumulation in the *Tetrahymen thermophila* ciliate. In addition, Tan*,* et al. [40] showed that Cd^+2^ and Zn^+2^ were heavily uptaken and retained in *Daphnia magna* when adsorbed to TiO_2_NPs.

Nigro*,* et al. [76] investigated the effect of single or combined exposure to Cd^+2^ and TiO_2_NPs for 7 days on various genotoxicity indicators in the European sea bass, *Dicentrarchus labrax.* Their results verified that individual Cd^+2^ and TiO_2_NPs exposure reduced genome template stability. The chromosome alteration was due to TiO_2_NPs exposure alone, although the damage to DNA was ineffectual; the opposite was seen in Cd^+2^ exposed specimens. On the other hand, joint exposure inhibits chromosomal damage and partially recovers the genome template stability.

Despite the lack of in vitro studies evaluating the outcomes of mutual exposure to Cd^+2^ and TiO_2_NPs in the cells of soil and aquatic organisms, in vitro studies have been performed using human and rodent cell lines. For instance, using human embryo kidney 293T (HEK293T) cells, Xia*,* et al. [35] evaluated the outcome of co-exposure to Cd^+2^ and TiO_2_NPs on the oxidative stress indicators, including the activities of catalase and superoxide dismutase enzymes and concentrations of ROS and malondialdehyde. The study findings verified that Cd^+2^ and TiO_2_NPs exerted synergistic effects on the cellular oxidative damage of HEK293T cells.

### 2.2. TiO_2_NPs and As Co-Exposure

As is an extremely toxic pollutant, highly detected in groundwater [77]. TiO_2_NPs have a high adsorption ability for As ions because of their large surface area and the presence of high-affinity hydroxyl surface groups [78]. For instance, it was reported that carp co-exposure to TiO_2_NP and As, either as As^+3^ or As^+5^, resulted in both As and TiO_2_NP accumulation in vital organs [46,47]. Higher amounts of TiO2 and As accumulated in the gills, stomach, and intestine compared to muscles. 

Wang, et al. [48] assessed the effect of TiO_2_NPs and As^+5^ interaction on *Ceriodaphnia dubia* and concluded that the decline in residual As^+5^ amounts decreases the toxic effect. In addition, the As^+5^ sorption onto the TiO_2_NPs surface adds to the toxicity once NPs enter the body. Additionally, Li*,* et al. [69] revealed that, at higher concentrations of TiO_2_NPs, As^+5^ adsorbed onto TiO_2_NPs could disassociate and lead to increased *D. magna* toxicity. In contrast, Yan*,* et al. [79] reported that TiO_2_NPs alleviated As^+5^ toxic effects in *Artemia salina nauplii* by increasing efflux and reducing As^+5^ amounts in the sensitive cellular fractions, including heat-sensitive proteins and organelles.

Nunes*,* et al. [49] evaluated the effect of TiO_2_NPs (1 mg/L) and As^+3^ (50 μg/L) co-exposure for 48 h on *Laeonereis acuta*, an estuarine polychaeta. Their findings revealed that TiO_2_NPs and As^+3^ mutual exposure increased ROS levels, decreased total antioxidant capacity, increased GR activity, and damaged macromolecules, including DNA, lipid, and protein. Moreover, the TiO_2_NPs and As^+3^ co-exposure affected the As metabolization capacity, leading to increased formation of a moderately toxic compound, known as dimethylated As. 

Luo*,* et al. [50] evaluated the effect of TiO_2_NPs exposure on the bioaccumulation and methylation of As in two freshwater algae (*Scenedesmus obliquus* and *Microcystis aeruginosa*), reared in water contaminated with inorganic As. The transmission electron microscope examination showed that TiO_2_NPs entered exposed algae. The TiO_2_NPs within the algae significantly increased As^+3^ and As^+5^ accumulation in *S. obliquus* and *M. aeruginosa*, respectively. *S. obliquus* was more sensitive than *M. aeruginosa* to As connected with TiO_2_NPs, thus, it has higher As methylation. 

Yang*,* et al. [80] explored the effect of TiO_2_NPs on the trophic transfer of As^+5^ from *Nannochloropsis maritima* microalgae to *Artemia salina nauplii* shrimp. The authors found that TiO_2_NPs considerably facilitated As^+5^ sorption on a *N. maritima* 24 h exposure period. This sorption promoted As trophic transfer from the algae to *A. salina*. Nevertheless, after depuration for 48 h, the As^+5^ retention in *A. salina* fed As^+5^ *-*TiO_2_NPs-contaminated algae was lower than that in *A. salina* fed As^+5^ -contaminated algae at equal exposure levels. This result demonstrates that the higher food chain transfer of As^+5^ in the presence of TiO_2_NPs can be elucidated by As^+5^ adsorption onto TiO_2_NPs in contaminated algae. Still, the As^+5^ bioavailability in *A. salina* is decreased with the NPs presence. 

Nunes*,* et al. [81] evaluated the consequences of combined exposure to two crystalline forms of TiO_2_NPs (rutile and anatase; 1 mg/L) and As^+3^ (50 μg/L) for 48 h on accumulation, metabolization, and toxicity of As^+3^ in the golden mussel *Limnoperna fortunei.* Results showed that both crystalline TiO_2_NPs forms affected the metabolization ability and enhanced more As^+3^ accumulation. In addition, TiO_2_NPs alone or in combination with As^+3^ induced oxidative stress in *L. fortunei* gills.

### 2.3. TiO_2_NPs and Cu Co-Exposure

Cu^+2^ is a frequently found metal ion in water and a vital micronutrient for aquatic organisms, but it may result in acute toxicity at high levels. Evidence shows that Cu^+2^ toxicity is associated with the interaction between adsorption of Cu^+2^ and co-substrates coordination [82]. The coexistence of NPs with Cu^+2^ raises concern about enhanced toxicity for Cu^+2^, even if at low levels.

A previous study revealed that the TiO_2_NPs coexistence with Cu^+2^ ion potentiated the toxicity of Cu^+2^ to daphnids even at low concentrations [51]. The metallothionein production in organisms is chiefly performed through the interaction of thiol groups with the HM. Hence, the authors speculated that the TiO_2_NPs could compete with sulfhydryl groups by adsorbing or binding free Cu^+2^ ions, which cause metallothionein detoxification to be inhibited. Additionally, in *Daphnia magna*, Fan*,* et al. [52] reported that Cu^+2^ increased oxidative stress and physiological damage in the presence of TiO_2_NPs due to Cu^+2^ sorption. In addition, TiO_2_NPs may have inhibited Na^+^/K^+^-ATPase by hindering the transfer channel of Na^+^/K^+^.

Rosenfeldt*,* et al. [53] investigated the effect of exposure to TiO_2_NPs (2 mg /L) and Cu^+2^ (40 g /L) for 24 days on the amphipod *Gammarus fossarum* mortality and health. In the presence of TiO_2_NPs, Cu^+2^-inducing toxicity was mostly eliminated. It was proposed that the Cu^+2^ toxicity reduction is linked to Cu^+2^ elimination from the water column through TiO_2_NPs agglomeration and sedimentation and the metal ions absorbed [83].

### 2.4. TiO_2_NPs and Pb Co-Exposure

Pb^+2^ is a heavy metal that has been used for thousands of years in the manufacture of human utilities. These include wine, pigments, glass, recipients, and, more recently, antiknock fuel additives, batteries, and electronic components [84]. Industrial, agricultural, and urban waste are key sources of Pb^+2^ release to the environment [85]. As such, Pb^+2^ is one of the major toxic pollutants [86].

Zhang*,* et al. [54] investigated the TiO_2_NPs (50 and 120 nm) and Pb^+2^ interaction in adult mice and verified that no synergistic interaction exists between TiO_2_NPs and PbAC in orally administered mice. Still, Pb^+2^ may increase the TiO_2_NPs acute toxicity to some extent. In human embryo hepatocytes, Du*,* et al. [36] confirmed that TiO_2_NPs (0.001, 0.01, 0.1, 1, and 10 μg/ mL) and Pb^+2^ (1 μg/ mL) in combination induced cytotoxicity and oxidative stress in the absence of photoactivation.

Miao*,* et al. [55] examined the effect of TiO_2_NPs (0.1 mg/L) and/or Pb^+2^ (0, 5, 10, 20, and 30 g/L) exposure for 6 days post-fertilization on nervous systems and thyroid function of zebrafish (*Danio rerio*) larvae. The results of their experiment suggested that TiO_2_NPs increase Pb bioconcentration, leading to the disturbance of the neuronal system and the thyroid function in zebrafish larvae. Additionally, in zebrafish larvae, Hu*,* et al. [87] examined the influence of TiO_2_ NPs on Pb^+2^ bioconcentration, depuration, and neurotoxic impacts. The results indicate that TiO_2_NPs may act as a Pb^+2^ carrier and augment its bioconcentration, although free Pb^+2^ concentration decreases due to the NP-Pb complex formation, thus decreasing toxicity to larvae.

Vicari*,* et al. [56] evaluated the effect of waterborne exposure of Neotropical fish species, *Hoplias intermedius*, to TiO_2_NPs (100 mg/L) and/or Pb^+2^ (0.033 mg/L) toxicity for 96 h. The authors verified that TiO_2_ NPs alone caused DNA damage in the cells of vital tissues, including the brain, gill, and kidney, but the muscle AChE activity reduced in the Pb-only exposed group. However, the metallothionein concentration was significantly increased in the TiO_2_NPs+Pb^+2^ co-exposed group.

Matouke and Mustapha [88] assessed the bioaccumulation profile of TiO_2_NPs and Pb^+2^ and their impacts on the copepods feeding behavior in a basic food chain, comprising the cyclopoids copepods (*Eucyclop* sp.) and freshwater alga *Chlorella ellipsoides*. The results showed that the TiO_2_NPs and Pb^+2^ mutual exposure impaired microalgae ingestion and filtration via cyclopoid copepods and increased antioxidant enzymes, lipid, and carbohydrate levels because of stress.

Oya-Silva*,* et al. [89] investigated the TiO_2_NPs and Pb^+2^ interaction effect on the biochemical and genetic biomarkers in the freshwater fish *Rhamdia quelen*. The study results showed that TiO_2_NPs alone and co-exposure of TiO_2_NPs and Pb^+2^ can produce significant short exposure toxic effects. 

## 3. Zinc Oxide Nanoparticles

**Figure 2:**
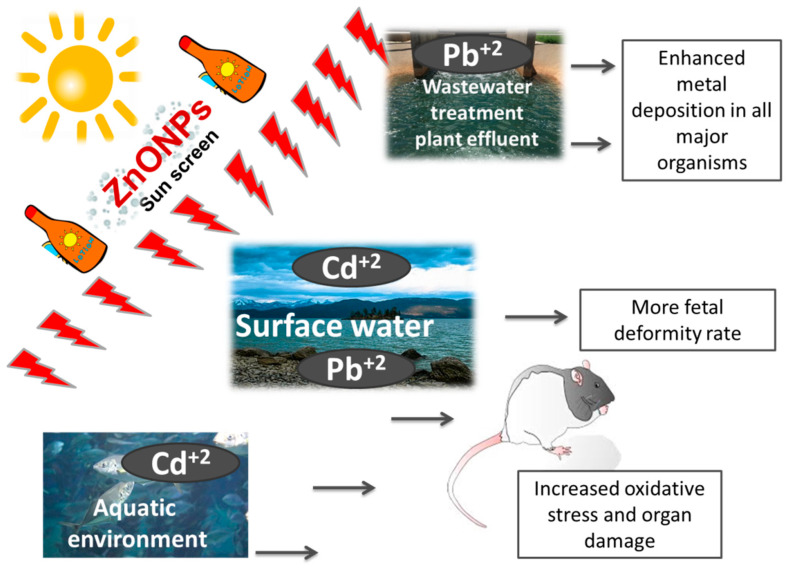
Zinc oxide nanoparticles interactions with different heavy metals.

Zinc oxide nanoparticles (ZnONPs) have been used extensively as sunscreens that can easily be released into the water [90]. Because of their small size and large surface areas, ZnONPs adsorb numerous environmental contaminants [70] (Figure 2). In wastewater treatment plant effluents, environmental ZnONPs concentrations were up to 45 μg/L [91]. Surface water ZnONPs concentrations were up to 74 μg/L in the United States [92] and 1.84 μg/L in Singapore [93]. The environmental level of ZnONPs is inevitably increased as production volumes and applications increase [94].

Jia*,* et al. [57] assessed the effect of oral administration of ZnONPs (14 or 58 nm) and/or Pb^+2^ at tolerable doses to healthy overweight and healthy normal weight mice. Compared to normal mice, the ZnONPs enhanced Pb^+2^ deposition in all major organisms in the overweight mice. In the overweight mice, higher levels of hepatic ROS, proinflammatory cytokines, and liver damage were found. These findings emphasized the potential increased risk of co-exposure of NPs/HM in the sensitive overweight population.

Teng*,* et al. [59] evaluated combined ZnONPs and Cd^+2^ toxicity using two oral-administered pregnant mouse models during peri-implantation or organogenesis. The authors verified that combined exposures to ZnONPs and Cd^+2^ resulted in a higher fetal deformity rate at the organogenesis stage rather than peri-implantation stage co-exposures. Moreover, after Cd^+2^ adsorption, the surface charge of ZnONPs was modified. The resulting nanoadducts caused the shedding of endothelial cells and damage to placental barriers. Furthermore, lower expression of tight junction proteins, including claudin-4, -8, and ZO1, was observed. These molecular and cellular events increased maternal-fetal transmission of both pollutants and worsened embryotoxicity.

Khayal, et al. [58] assessed the effect of joint oral exposure of ZnONPs (85 mg/kg b.wt.) and (Pb^+2^ 10 mg/kg b.wt.) for 8 weeks on the thyroid gland of adult rats compared to their individual exposure. The results revealed that the mutual exposure of ZnONPs and Pb^+2^ resulted in greater thyroid dysfunction represented by reduced serum levels of triiodothyronine and tetra-iodothyronine but increased thyroid-stimulating hormone levels. In addition, the concentrations of Zn^+2^ and Pb^+2^ in the serum and thyroid were greater in the ZnONPs and Pb^+2^co-exposed rats.

## 4. Silica Nanoparticles (SiNPs)

SiNPs are widely utilized in biomolecular detection, medication delivery, imaging, diagnosis, photodynamic therapy, and gene therapy [95,96]. SiNPs are the most found NPs in the atmosphere [97,98], while Pb^+2^ is another known toxic air pollutant from anthropogenic activities. Feng*,* et al. [61] evaluated the effect of mutual exposure to SiNPs (2 mg/kg b.wt.) and PbAc (0.25 mg/kg) for 30 days on the hearts of males Sprague Dawley rats. The authors verified that combined exposure to SiNPs and Pb^+2^ could worsen cardiovascular toxicity through hypercoagulation, endothelial damage, and cardiac injury.

Guo*,* et al. [60] showed that the intraperitoneal administration of SiNPs (20 mg/kg BW) and CdCl_2_ (1.5 mg/kg BW) in mice once a day for seven successive days significantly increased Cd^+2^ biodistribution density in the kidney and liver but did not alter the Si distribution in all examined organs. In addition, SiNPs and Cd^+2^ co-exposure resulted in greater severe oxidative stress in the renal and hepatic tissues.

## 5. Aluminum Oxide Nanoparticles

Al_2_O_3_NPs are among the most common ENMs, with various industrial and biomedical applications, including cutting tools, packaging materials, refractory products, semiconductor materials, and cosmetic fillers [99]. Its elemental shape (nano-Al) is also an important military material that provides the basis for a higher fuel for space launch vehicles [100]. Despite their wide use, numerous studies have displayed that individual Al_2_O_3_NPs exposure was accompanied by increased ROS production, mitochondrial dysfunction, protein damage, and impaired cell morphology [101]. Additionally, exposure to Al_2_O_3_NPs may lead to harmful effects, including genetic and DNA damage [102], apoptotic consequences [103], inflammatory reactions [104], and carcinogenicity [105].

Due to their strong affinity to As^+5^, Al_2_O_3_NPs products commonly remove As from drinking water [106,107,108]. These applications could cause high environmental releases of Al_2_O_3_NPs and As mixture. Hence, the possible effects of Al_2_O_3_NPs and As co-exposure on the ecosystem have gained researchers’ attention. For instance, Wang, et al. [62] demonstrated that the combined exposure of *Ceriodaphnia dubia* to Al_2_O_3_NPs and inorganic As^+5^ resulted in an enhanced toxic effect, as As^+5^ was adsorbed on the Al_2_O_3_NPs surface.

## 6. Discussion

NPs are released into the environment in large amount due to being extensively used and may affect the toxicity of other pollutants already found in the environment, such as HMs [34]. Hence, studying the outcomes of co-exposure to NPs and HMs on the non-target organisms is considered an important issue for the proper evaluation of the hazards of NP use. Several studies have evaluated the effects of mutual exposure of different NPs with their highly expected co-occurred HMs in the environment. Interestingly, different outcomes from the NPs and HMs co-exposure were recorded. In some experiments, reduced toxic effects of HMs was obvious at co-occurrence with NPs [34], while on the other hand, some NPs increased HMs toxicity [55,109]. Moreover, in some cases, the response of the living organisms to the combined exposure to NPs and HMs did not differ from that of the single exposure to each of them [34]. Several factors have been found to affect the outcomes of NPs and HMs mutual exposure, such as the diameter of NPs, the NPs crystal structure, the species of living organisms, and the exposure media. For instance, 30 nm TiO_2_NPs increased the Cd^+2^ toxicity to the freshwater green alga, *Pseudokirchneriella subcapitata*, but 300 nm TiO_2_NPs reduced the Cd^+2^ toxicity to the same algae species [110]. Additionally, the Cr (VI) toxicity to *Scenedesmus obliquus* substantially reduced in the presence of TiO_2_NPs anatase [111], while rutile TiO_2_NPs significantly increased the Zn^+2^ toxicity to *Anabaena* sp. [112]. Moreover, 30 nm TiO_2_NPs reduced Cd^+2^ toxicity to *C. reinhardtii* [34] but increased the Cd^+2^ toxicity to *P. subcapitata* [110]. Of note is the characteristic of the exposure media, in which NPs and HM present could affect their toxicity, such as pH, the dissolved organic matter (DOM) amount, and the presence of complexing agents, such as thiosulfate or chloride [113,114,115]. For instance, the presence of NPs in media with high pH and DOM has been reported to reduce its toxicity potential [116]. The presence of DOM may decrease the toxicity of NPs by promoting the formation of NPs-DOM complexes [116]. These complexes may present coating on NPs, thus blocking oxidation sites and decreasing the release of free ions from the material [117]. The free ions are known as the driver for NP toxicity [118]. In addition, the presence of DOM could decrease the NP toxicity by decreasing the free ions availability because of the NPs-DOM complexes formation [114]. The alkaline environments have been reported to increase the toxicity of some HMs, such as Cd ^+2^ [115]. The competition between the HM ions and protons at the cell surface could be responsible for the direct relationship between pH and HM toxicity [115,119]. Furthermore, low pH may initiate certain physiological reactions within organisms, such as metallothionein induction, limiting the HM toxicity [120]. Hence, further investigations on the impacts of the exposure media factors on the toxicity of co-occurred HMs and NPs and on the different organisms are highly needed.

The outcomes of joint HM and NP exposure may be affected by the duration of exposure. It is well known that, in reality, species can be exposed to contaminants not only throughout their entire life but also over many generations, which may have a greater impact on the population [121]. Thus, despite many short-term studies assessing NPs and/or HMs exposure outcomes [69,79,80], several studies have evaluated the impacts of the individual or mutual NP and HM long-term or multigenerational exposure [122,123,124,125]. It was evident that the outcomes of the co-exposure to NPs and HMs during the parental generation can affect the health of offspring in various ways. Some reports confirmed that maternal exposure to NPs amplifies the multigenerational HMs toxicity by promoting the HMs accumulation in germ cells [20]. Other reports verified the reduction of toxicity of HMs at parental co-exposure with NPs because of the NP-induced alteration of the HMs metabolism in the gut [125]. Thus, it is necessary to perform more research to elucidate the factors that control the outcomes of NP and HM co-exposure on the multigenerational soil and aquatic organisms. 

Several mechanisms could be underlying the synergistic toxic effects due to the co-exposure of NPs and HMs. The first mechanism involves the internalization of NPs within cells, releasing toxic concentrations of HMs [126]. Initially, NPs have a large surface area that adsorbs the HMs [127,128]. Then, NPs may act as carriers for the HM transport within the organism; the HMs can enter the organism as a free ion and/or NPs-HMs complex [129]. The complexed contaminants can then be released inside the organisms. The earlier mechanism largely depends on NPs stability as some NPs are unstable and rapidly release the adsorbed HMs free ions, inducing cytotoxic effects [34]. In addition, the existing NPs-HMs complex may interact with intra and extracellular molecules, forming coronas, altering their biological activity. Furthermore, the NPs themselves may de-aggregate and release their core ions, e.g., Ag^+2^ and AL^+3^, and cause toxicity rather than the HMs that are adsorbed to the NPs [70]. Through the mechanism above, several NPs have been proved to increase the HMs bioavailability and toxicity [47,57,124]. The second mechanism depends on the NPs ability to alter the HMs speciation and bioavailability, consequently increasing toxicity [70]. For instance, TiO_2_NPs and As II co-exposure to worm *Laeonereis acuta* influenced the As metabolization capacity via increasing dimethylated As, a moderately toxic form, damaged lipids, and DNA [49]. The third mechanism is related to the NP ability to increase HM toxicity through altering biotic ligands availability [70]. In this regard, some NPs have been reported to form complexes with cations in the exposure medium, reducing the competition of binding between hard cations and HM free ions on the biotic ligand and increasing the HM bioaccumulation and lethality to organisms [130]. The fourth mechanism involves the ability of NPs to disrupt the cell membrane and increase HM uptake [70]. By the same mechanism, several NPs increased the HM toxicity in in-vitro models [131,132].

On the other hand, the amelioration of toxicity of HMs at the co-exposure with NPs could be associated with the potent antioxidant activity of some NPs [34,111]. In addition, the higher electrostatic attraction of the NPs to the surface of some living organisms compared to HM or NP-HM complexes could reduce the HM internalization within the organisms [42]. For instance, Yu*,* et al. [34] reported that the negatively charged TiO_2_NPs and SiO_2_NPs surfaces were responsible for their adsorbance to the algal cell surface. At the same time, a minute amount of NP-Cd complexes were adsorbed, and the rest was aggregated and sedimented in the surrounding environment. Subsequently, both the soluble Cd^+2^ concentration around algae and the Cd^+2^ internalization were reduced. Additionally, Dalai*,* et al. [111] revealed that the reduced Cr^+6^ toxicity to *Scenedesmus obliquus* in the presence of TiO_2_NPs was mainly linked to the Cr^+6^ adsorption on the TiO_2_NPs surface, resulting in its aggregation and precipitation.

Overall, the NP and HM co-effects are a complex problem. Various aspects still need to be covered, such as the environmental factors and species differences determining the positive or negative outcomes of NP and HM mutual exposure. Consequently, experimentally developing proper test designs for evaluating such mixture effects is considered a huge challenge. In addition, the ability to standardize test guidelines for the testing of these mixtures is another important challenge. The appropriate test design should consider several factors, such as the exposure media effect, NPs aggregation behavior in the test medium, the physical interactions between NPs and HMs, the physical interactions between NPs and organisms, the possible cell membrane disruption, and the potential free metal ions release. In addition, due to the growing number and variety of NPs, e.g., carbon-based NPs, other factors to be considered for the development of test designs include NP size, shape, and even surface functionalization [123]. Moreover, long-term exposure studies are required, particularly with the continual uptake and accumulation of NPs and HMs in the tissues of the living organisms. Furthermore, future work aimed at explication of the multigenerational effects of pollutants associated with NPs is highly needed. 

## 7. Conclusions

As production of ENMs increases rapidly, the potential NPs eco-toxicity impacts cause global concern. The reviewed studies’ collective findings underline that studying the potential interactions of NPs with existing environmental pollutants are vital in evaluating the possible NPs environmental risks. Identifying the expected hazards of co-exposure to ENMs and environmental contaminants could greatly help determine the safe strategies for combating such hazards. This could benefit the health of the different living organisms, environment sustainability, industrial companies, and international standardization organizations.

## Figures and Tables

**Table 1:** Outcomes of co-exposure to engineered nanomaterial and heavy metals in different experimental models.

Tested Conditions	Nanoparticle	Heavy Metal	Tested Organism	Co-Exposure Outcome	Refs.
**I. In vitro models**	TiO_2_NPs	Cd^+2^	Human embryo kidney 293T (HEK293T) cells	Cd^+2^ and TiO_2_NPs exert synergistic effects on the cellular oxidative damage of HEK293T cells	[35]
		Pb^+2^	Human embryo hepatocytes	TiO_2_NPs and Pb^+2^ in combination induced cytotoxicity and oxidative stress in the absence of photoactivation.	[36]
**II. In vivo models**	TiO_2_NPs	Cd^+2^	Carp (*Cyprinus carpio*)	A positive correlation was found between Cd^+2^ and TiO_2_NPs concentrations.	[37]
			The Mediterranean mussel *(Mytilus galloprovincialis*)	TiO_2_NPs and Cd^+2^ co-exposure did not increase adverse effects in *M. galloprovincialis*.	[38]
			Zebrafish (*Danio rerio*)	The presence of TiO_2_ NPs with Cd^+2^ slightly increased the uptake rate constants of Cd^+2^ in fish	[39]
			Water flea *(Daphnia magna*)	TiO_2_NPs the uptake and retention of Cd^+2^	[40]
			Water column crustacean *Daphnia magna* Sediment *oligochaete Lumbriculus variegatus*	TiO_2_NPs increased the total Cd^+2^ body burden, but no change in toxicity was observed.	[41]
			*Chlamydomonas reinhardtii*	TiO_2_ NPs presence alleviated the Cd^+2^ toxicity	[42]
			Water flea *(Daphnia magna*)	TiO_2_NPs transport Cd^+2^ and Zn^+2^ into *D. magna*.TiO_2_NPs provide potential adsorption binding sites for Cd^+2^ within the *D.magna* gut.	[43]
			The ciliate *Tetrahymena thermophila*	TiO_2_NPs enhanced Cd^+2^ accumulation	[44]
			Asian clam *(Corbicula fluminea)*	The presence of TiO_2_NPs did not affect Cd^+2^ uptake by *C. fluminea*.	[45]
		As	Carp (*Cyprinus carpio*)	TiO_2_NPs increased As^+5^ concentrations and bioavailability	[46,47]
			Water flea *(Ceriodaphnia dubi*a)	As^+5^ sorption onto the TiO_2_NPs surface contributes to the toxicity once nanoparticles enter the body.	[48]
			*Laeonereis acuta*	TiO_2_NPs and As^+3^ co-exposure synergistically toxic	[49]
			Freshwater algae *(Microcystis aeruginosa and Scenedesmus obliquus)*	TiO_2_NPs boosted As^+3^ and As^+5^ accumulation and methylation	[50]
		Cu^+2^	Water flea *(Daphnia magna)*	The coexistence of TiO_2_NPs with Cu^+2^ enhances the toxicity of Cu^+2^ to daphnids even at low concentrations	[51]
			Water flea *(Daphnia magna)*	Cu^+2^ in the presence of TiO_2_NPs induced higher levels of oxidative stress and physiological damage	[52]
			The leaf shredding amphipod *Gammarus fossarum*	The presence of TiO_2_NPs largely eliminated Cu^+2^-induced toxicity.	[53]
		Pb^+2^	Mice	No synergistic interaction exists between TiO_2_NPs and Pb^+2^.	[54]
			Zebrafish *(Danio rerio)* larvae	TiO_2_NPs increase bioconcentration of Pb^+2^	[55]
			Neotropical fish species *Hoplias intermedius*	TiO_2_NPs induced oxidative stress increase at co-exposure with Pb^+2^	[56]
	ZnONPs	Pb^+2^	Mice	ZnONPs enhanced the deposition of Pb in all major organs in the overweight mice	[57]
		Pb^+2^	Rat	The joint exposure of Pb^+2^ and ZnONPs resulted in an additive toxic effect on the thyroid gland	[58]
		Cd^+2^	Mice	Combined ZnONPs and Cd^+2^ exposures at the organogenesis stage induced higher fetal deformity	[59]
	SiNPs	Cd^+2^	Mice	Synergistic effect of SiNPs and Cd^+2^	[60]
		Pb^+2^	Sprague Dawley male rats	Co-exposure to SiNPs and Pb^+2^ resulted in additive and synergistic effects on the cardiovascular system.	[61]
	Al_2_O_3_NPs	As^+5^	*Ceriodaphnia dubia*	Al_2_O_3_NPs and inorganic As^+5^ co-exposure resulted in enhanced toxic effect	[62]

Al_2_O_3_NPs: aluminum oxide nanoparticles; As: arsenic; Cd: cadmium; Cu: copper; Pb: lead; SiNPs: Silica nanoparticles; TiO_2_NPs: titanium dioxide nanoparticles; ZnONPs: Zinc oxide nanoparticles.

## Data Availability

All datasets generated for this study are included in the article.
